# Cancer cells evade ferroptosis: sex hormone-driven membrane-bound O-acyltransferase domain-containing 1 and 2 (MBOAT1/2) expression

**DOI:** 10.1038/s41392-023-01593-3

**Published:** 2023-09-08

**Authors:** Alexia Belavgeni, Wulf Tonnus, Andreas Linkermann

**Affiliations:** 1 Division of Nephrology, Department of Internal Medicine III, University Hospital Carl Gustav Carus at the Technische Universität Dresden, Dresden, Germany; 2 Division of Nephrology, Department of Medicine, Albert Einstein College of Medicine, Bronx, NY USA

**Keywords:** Cell biology, Cancer metabolism

In a recent manuscript published in *Cell*, Liang et al. discovered novel ferroptosis regulators, membrane-bound O-acyltransferase domain-containing 1 and 2 (MBOAT1/2).^1^ These findings may have implications for ferroptosis-based cancer therapy.

Ferroptosis is a form of iron-catalyzed necrotic cell death that is permanently prevented in most cells by two surveillance systems. The best investigated ferroptosis inhibitory mechanisms are glutathione peroxidase 4 (GPX4) and glutathione-independent ferroptosis-suppressor protein 1 (FSP1). While GPX4-deficient mice die from ferroptosis in highly sensitive tissues, such as kidney tubules,^2^ FSP1-deficient mice are viable and fertile.^3^

Over the last decade, strong evidence has emerged for ferroptosis as a key driver of cardiovascular complications, such as myocardial infarction, stroke, and acute kidney injury.^4^ Therefore, a pharmacological strategy to inhibit ferroptosis would make sense and is currently being tested by several pharmaceutical companies. In contrast, cancer cells and virally infected cells have been demonstrated to evade host ferroptosis, e.g., by stabilizing and overexpressing the above-mentioned anti-ferroptotic systems or by expressing anti-ferroptotic peptides such as vPIF-1. Specifically targeting cancer cells for ferroptosis, therefore, is another important therapeutic strategy. However, it is unclear to what extent ferroptotic cell death supports or inhibits cancer immunotherapy. Given that thousands of manuscripts have been published on ferroptosis in the last 10 years, it would be considered a major breakthrough to identify novel ferroptosis-inhibiting mechanisms that function independently of GPX4 and FSP1.

**Fig. 1:**
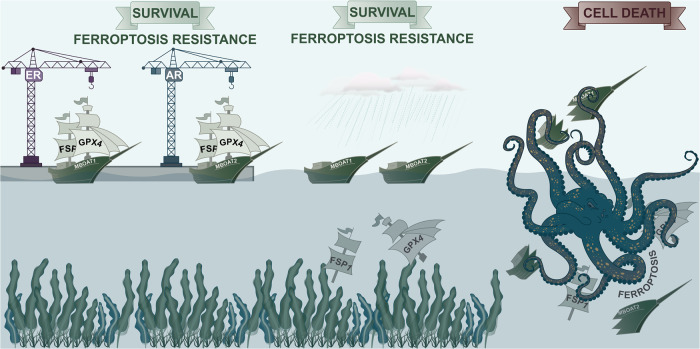
MBOAT1/2 prevents cell death of cancer cells that are deficient in the two major ferroptosis-surveillance systems GPX4 and FSP1. For cancer cells to proliferate (to sail the open seas) they must prevent ferroptosis (evade the ferroptopus). Ferroptopus is derived from “ferroptosis” and “octopus” which means “Ferroptosis is like an octopus”. Several ferroptosis-surveillance systems biologically emerged. Most cells rely on glutathione peroxidase 4 (GPX4) and the ferroptosis-suppressor protein 1 (FSP1) to survive (in our little cartoon, FSP1 and GPX4 are the masts of the boats). In addition to GPX4 and FSP1, prostate cancer cells produce MBOAT2 downstream of the androgen receptor (AR, the AR shipyard). In contrast, breast cancer cells generate MBOAT1 downstream of the estrogen receptor alpha (ERα, the ER shipyard). MBOAT1 and MBOAT2 can still prevent capsize in the absence of GPX4 and FSP1 (i.e., in stormy weather if the two masts of the ships are destroyed). Only if the keel of MBOAT1 or MBOAT2 breaks, cancer cell death by ferroptosis occurs (death by water influx caused by the ferroptopus)

Liang et al. investigated GPX4-deficient cells on the HT1080 cell background, a default cell line for ferroptosis research. In two parallel positive selection screens for resistance to a covalent GPX4-inhibitor (RSL3) and cysteine starvation, respectively, they identified seven sgRNAs that promoted survival in both of these screens. Five of them were known regulators of ferroptosis, but two were novel: MBOAT2, a lyso-PL acyltransferase, and a secreted phospholipase A2 family member (PLA2G2F). Next, they generated inducible GPX4-FSP1-double deficient HT1080 cells and demonstrated that overexpression of MBOAT2, but not PLA2G2F, was capable of rescuing these cells from ferroptosis as demonstrated by crystal violet staining. Their further work on MBOAT2 showed that it requires mono-unsaturated fatty acids (MUFAs) of any source (endogenous or exogenous) to function. As expected, MBOAT2-dependent inhibition of ferroptosis is associated with phospholipid remodeling. Similar results were obtained when MBOAT1, a related acyltransferase, was investigated. While MBOAT2 was upregulated in prostate cancer cells upon androgen receptor (AR) activation, MBOAT1 is regulated downstream of the estrogen receptor alpha (ERα) in breast cancer cells. In both cases, interference with the respective receptor by anti-AR drugs (enzalutamide) or ER-degraders (fulvestrant) antagonizes MBOAT expression and results in much lower tumor volumes in mouse models, demonstrating pathophysiological relevance. In conclusion, the MBOAT system can keep cancer cells alive even if GPX4 and FSP1 are absent or dysfunctional (Fig. 1).

An increasing body of evidence suggests that steroid hormones are central regulators of several ferroptosis-regulating systems. While androgens and estrogens have been demonstrated in this study, other hormone-producing cancers, such as adrenocortical carcinomas, are highly sensitive to ferroptosis. Even commonly used synthetic steroids, such as dexamethasone, a blockbuster anti-cancer drug, sensitize to ferroptosis in vivo.^5^ Dexamethasone, despite its unknown mechanisms of action on cancer cells, might well owe parts of its success to this pro-ferroptotic activity. We therefore cannot help but hypothesize that hormone-producing cells are particularly prone to ferroptosis. This may soon become clinically relevant as ferroptosis-inducers of all types are entering clinical trials. Importantly, hormone monitoring is not routinely included in the classical cancer trial design but potential side-effect profiling during these trials should consider the endocrine ferroptosis susceptibility.

At last, the relation between sex hormones and ferroptosis regulation by MBOATs is falling into place. It has been known for at least half a century that cardiovascular complications are less pronounced in females. Today, we understand that the bulk of cell death in a heart attack, acute kidney injury or a stroke is mediated by ferroptosis. It will be of importance to identify the precise mechanisms of the sex difference in ferroptosis sensitivity. The eminent work by Liang et al.^1^ sets the stage and focuses the limelight on ferroptosis as a prototype research area for studying sex differences.
